# Validation of a Dried Blood Spot Assay for Testosterone Measurement Using Liquid Chromatography‐Tandem Mass Spectrometry

**DOI:** 10.1002/ansa.202400035

**Published:** 2024-11-02

**Authors:** Yehudah Gruenstein

**Affiliations:** ^1^ Department of Biochemistry and Molecular Biology University of Miami Coral Gables Florida USA

**Keywords:** assay validation, dried blood spot (DBS), hematocrit effect, liquid chromatography‐mass spectrometry (LC‐MS/MS), testosterone

## Abstract

Testosterone is a critical hormone involved in regulating various physiological processes in both men and women. Accurate testosterone measurement is essential for diagnosing endocrine disorders such as hypogonadism and polycystic ovary syndrome and for routine testing. Traditionally, testosterone levels are measured using serum or plasma samples, which present challenges in sample collection, storage, and transport, particularly in resource‐limited settings. Dried blood spot (DBS) sampling has emerged as an effective alternative for hormone analysis, offering significant advantages in terms of sample stability, ease of collection, and simplified logistics. This study aimed to validate a DBS‐based testosterone assay using liquid chromatography‐tandem mass spectrometry (LC‐MS/MS) to ensure accuracy and precision comparable to conventional serum‐based methods. Drops of whole blood samples were collected from adult volunteers using a single‐use safety lancet for finger pricks, with blood applied onto DBS cards (PerkinElmer 226 Spot Saver RUO card) for further analysis. The testosterone was extracted from DBS using a liquid‐liquid method and analyzed with LC‐MS/MS. The assay demonstrated excellent linearity across a wide concentration range (0.1–100 ng/mL) with a correlation coefficient (r^2^) of 0.999 and achieved a lower limit of detection of 0.058 ng/mL and a lower limit of quantification of 0.086 ng/mL. The method showed high precision, with intra‐ and inter‐day coefficients of variation below 10%, and satisfactory recovery rates. Hematocrit correction and matrix effect evaluations confirmed the robustness of the assay for clinical and research applications. Additionally, the assay displayed a strong clinical correlation between testosterone levels in DBS and venous serum samples, supporting its reliability for testosterone monitoring. This validation study supports that the DBS‐based LC‐MS/MS testosterone assay is a reliable tool for testosterone quantification for routine testing.

## 1 INTRODUCTION

Testosterone, a key androgen hormone, plays a crucial role in regulating numerous physiological functions in both men and women, including reproductive health, muscle mass, bone density, and overall well‐being [1, 2]. Accurate and reliable measurement of testosterone levels is essential in clinical settings, particularly for diagnosing conditions such as hypogonadism, polycystic ovary syndrome, and other endocrine disorders [1, 3]. Traditionally, testosterone measurements are performed using serum or plasma, which are well‐established methodologies but present several logistical challenges, particularly for sample collection, storage, and transport. These difficulties become more pronounced in resource‐limited settings or in large‐scale epidemiological studies. To address these limitations, alternative methods such as dried blood spot (DBS) sampling have gained significant traction in recent years for hormone analysis, including testosterone [4, 5, 6].

Over the past decade, DBS sampling has been increasingly used for microsampling across various fields due to its significant advantages regarding ease of sample retrieval, shipment, and enhanced analyte stability [7]. DBS involves collecting a small drop of capillary blood from a simple and minimally invasive finger prick, which is then applied onto specialized filter paper. Compared to venous blood sampling, the DBS technique simplifies the process and eliminates the need for a trained medical professional such as a doctor or nurse. Patients or researchers can perform the sampling themselves with proper instruction. The technique also offers numerous benefits over traditional blood collection methods: it is less expensive, easier to transport, reduces storage space, and allows for increased stability of many analytes, including testosterone, which is critical in both clinical and research settings [8]. While DBS has been extensively validated for other analytes including drugs of abuse [9] and other steroids [7], there are relatively few studies focused on testosterone [4] quantification, as it presents unique challenges. The small volume of blood collected via DBS can lead to variability in results due to factors like hematocrit (HCT) differences, recovery efficiency, and the need for highly sensitive instrumentation. Furthermore, testosterone extraction and quantification from DBS require optimized processes to achieve the accuracy and precision of traditional serum‐based methods.

A major challenge in DBS sampling is the ‘HCT effect’ which occurs when the blood's HCT level influences the spreading of the blood on the filter paper, leading to uneven distribution and potential assay bias [10]. To mitigate this effect, strategies have been proposed that either correct for HCT or accurately determine the volume of blood in the DBS. While devices are available to ensure controlled collection of blood spots, these devices can be costly and require strict patient compliance, which diminishes the simplicity and cost‐effectiveness of the DBS method. Conversely, correcting for HCT allows for the continued use of traditional low‐cost DBS cards, although this typically requires the determination of HCT through venous blood sampling, which somewhat negates the advantages of DBS. Optical HCT detection modules, which can scan the DBS surface to estimate HCT levels, offer a potential solution [10]. In this study, HCT correction was achieved through the collection of a separate venous sample.

Recent advancements in analytical techniques, particularly liquid chromatography‐tandem mass spectrometry (LC‐MS/MS), have significantly enhanced the sensitivity and specificity of testosterone assays, including those using DBS [4, 11]. However, a standardized approach to DBS assay validation for testosterone measurement for routine testing remains necessary. This article has explored the key insights gained from DBS assay validation for testosterone measurement, addressing the opportunities for standardizing this method in clinical and research applications.

## 2 Materials and Methods

### 2.1 Specimen Collection

Drops of whole blood samples were collected from eight female and 12 male adult volunteers using a single‐use safety lancet for finger pricks. After the prick, a drop of blood was directly applied to the sampling paper/card within a pre‐marked circle, ideally one drop per spot. In this study, the PerkinElmer 226 Spot Saver RUO card, which has five pre‐marked circles, was used. It was important to avoid touching the circle area, especially before the blood was applied and fully dried. The predefined circle had to be filled homogeneously and symmetrically, with both sides of the card/paper showing the same red color. A minimum of one complete circle was required from each individual who participated in the study. Samples that showed signs of contamination, hemolysis, or insufficient volume were deemed unsuitable.

### 2.2 Specimen Transportation and Storage

Blood spots were completely dried before storage or transportation. In general, a minimum drying time of 1–2 h in an open space at room temperature (15–22°C) was recommended. Moisture could affect the quality of the blood samples on the card/paper. Therefore, after drying, DBS samples were protected from humidity and moisture by covering them with a paper overlay and packing them in low gas‐permeable zip‐closure bags with desiccant packages. DBS samples packed as described were transported through the mail in a high‐quality bond envelope. DBS cards received in the lab were stored in their original envelope at 4°C until analysis, or for up to 30 days. If longer storage was needed, the cards were stored at −20 or −70°C for up to 6 months. DBS extracts in 96‐well plates were stored in the sample injector, covered, at 10°C for 1 week. If longer storage was required, plates were stored at −20°C for up to 1 month [12].

### 2.3 Method of Testing

**FIGURE 1:**
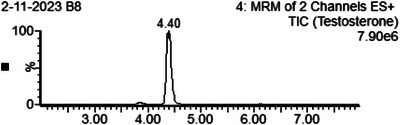
Sample chromatogram of testosterone. This figure illustrates the chromatogram for testosterone obtained using liquid chromatography‐tandem mass spectrometry (LC‐MS/MS) in electrospray ionization mode (ESI+). The sample was analyzed with a retention time of 4.40 min, and the chromatogram shows a total ion count (TIC) of 7.90e6. The MRM (multiple reaction monitoring) method was used, focusing on two channels to monitor testosterone ions. The sharp peak at 4.40 min indicates the separation and detection of testosterone with high specificity and sensitivity, confirming the successful quantification of the hormone in the sample.

The testing method utilized was LC‐MS/MS. LC separated the analytes of interest based on chromatographic retention, while MS determined the analytes’ molecular masses based on their mass‐to‐charge ratios. The principal steps in this measurement procedure were: extraction of the analyte from the sample matrix and quantitation of the analyte by isotope dilution high‐performance LC‐MS/MS using stable isotope‐labeled internal standards and calibrators. Isolation of the analyte was achieved using liquid‐liquid extraction. MS/MS was performed with a triple quadrupole mass spectrometer using ESI in positive ion mode. The analyte was identified based on chromatographic retention time (Figure 1) and on specific mass‐to‐charge ratio transitions using selected reaction monitoring [13].

### 2.4 Key Instruments

This study utilized a Waters Xevo TQ‐XS MS Detector for the LC‐MS/MS analysis. The system included key components such as a Column Manager, Acquity UPLC HSS T3 1.8 µm UPLC Column, Binary Solvent Manager, Sample Manager, and Sample Organizer, all sourced from Waters Corporation. Supporting auxiliary instruments included a PerkinElmer DBS Puncher, a Peak Scientific Nitrogen Generator, a Benchmark Scientific Heated Microplate Vortexer, and a Hanchen Electronic Scale. Additional devices like a Benchmark Scientific Centrifuge and a Co‐Z Sonicator were also employed for sample processing. For further details on specific models and serial numbers of the devices and consumables (refer to Tables S1–S3).

### 2.5 Reagents and Chemicals

The testosterone standard solution (Cerilliant USA, T‐037) was stored at −20°C and used to prepare calibration curves and quality control (QC) materials. The internal standard, a Carbon‐13 labeled testosterone (Cerilliant USA, T‐070), was prepared and used to assess recovery and precision in the assay. Ammonium fluoride (Sigma Aldrich, 338869) and LC‐MS‐grade methanol (Thermo Fisher) were used as mobile phases and solvents. Further details on the reagents and chemicals can be found in Table S4.

### 2.6 Sample Preparation

Testosterone standard and internal standard solutions were prepared via serial dilution. The testosterone standard solution (1 mg/mL) was diluted to create intermediate (1000X) and working (10X) solutions for spiking steroid‐free blood. Similarly, an internal standard solution was prepared with a dilution of 5 µg/mL (100X) to create a working solution (1X). Steroid‐free whole blood was prepared using blood collected from healthy volunteers. After centrifuging whole blood to separate plasma, the red blood cells (RBCs) were washed with phosphate‐buffered saline (PBS) three times and then mixed with steroid‐free serum to achieve a 40% HCT. These prepared blood samples were used to spot DBS cards with known concentrations of testosterone to generate calibration standards and QC samples. A detailed instruction protocol can be found in the sample preparation (A–E) section of the Supporting Information File.

### 2.7 DBS Extraction Protocol

Blood spots were prepared for extraction using the PerkinElmer DBS puncher. Three 3 mm discs from each blood spot sample were punched out in a 96‐well plate, and 10 µL of internal standard solution (10 ng/mL, 1X) was added to each well. Afterward, 500 µL of LC‐MS‐grade methanol was added, and the plate was sealed with adhesive film. The plate was shaken for 5 min at 1000 rpm, sonicated at 30°C for 20 min, and centrifuged at 2000 × *g* for 2 min. The supernatant was transferred to a separate plate and evaporated to dryness under nitrogen at 60°C. The dried samples were reconstituted with 100 µL of a 50:50 methanol/water solution and shaken for 10 min at 1000 rpm before centrifugation. The samples were then sealed for the injection procedure. A step‐by‐step protocol can be found in the operational procedure section (A) of the Supporting Information File.

### 2.8 Instrumental Start‐up and Operation

LC‐MS/MS analysis was performed under specific conditions, using ammonium fluoride as the mobile phase A and LC‐MS‐grade methanol as mobile phase B, with a flow rate of 0.8 mL/min. The column temperature was maintained at 50°C, and the sample compartment was set to 15°C. MS analysis employed positive ESI, with parameters including a capillary voltage of 1.0 kV and source temperature of 150°C. Multiple reaction monitoring transitions for testosterone and its internal standard were used to achieve high sensitivity and specificity in the measurements. See the Supporting Information File operational procedure section (B and C) for details.

### 2.9 Results Processing

Each sample run included an eight‐point calibration curve, ranging from 0 to 10 ng/mL, along with QC samples at low, medium, and high concentrations. Calibration curves were generated by plotting area ratios and required a coefficient (r^2^) of >0.98 to be considered valid. Calibration standards were required to fall within ±20% of their assigned values. For QC, two out of three QC points had to be between 80% and 120% of the assigned values. Samples were considered valid if the testosterone signal matched the predicted retention time and the internal standard signal aligned within 15% of the calibration curve; otherwise, they were considered inconclusive. See the result interpretation section in the Supporting Information File for details.

### 2.10 Validation Methods

The method was validated by assessing interference, measuring range, the lower limit of quantification (LLOQ), the limit of detection (LOD), accuracy, precision, carryover, and clinical correlation between DBS and venous serum testosterone concentrations. Additionally, method performance related specifically to DBS was evaluated, including the effects of HCT, blood spot volume, matrix effects, recovery, and stability.

- Interference:

Interference from other steroids was assessed by adding 11 different steroids to a standard testosterone sample and checking for possible chromatographic coelution and MS detection of the other steroid hormones.

b. Measuring Range:

Testosterone was spiked at various concentration levels and a calibration curve was established with seven concentration levels. A linear calibration model was generated (peak area ratio of each steroid to its respective IS) using a 1/x weighted least‐squared regression. The range was considered linear (Figure 3) if the determination coefficient was greater than 0.99 and in the absence of a systematic pattern in the residuals. The reportable range of results is the range in which linearity was verified.

c. Lower LOQ:

LLOQ refers to the lowest level analyte that can be determined with acceptable performance. The LLOQ was calculated as follows: Mean of 10x DBS Blank + 10 SD = 0.0046 + 10 × 0.004 = 0.086 ng/mL.

d. Limit of Detection:

LOD refers to an analyte level that is significantly different from zero.

The LOD was calculated as follows: Mean of 10x DBS Blank + 3 SD = 0.046 + 3 × 0.004 = 0.058 ng/mL.

e. Accuracy:

The accuracy of the DBS LC‐MS/MS method was evaluated using QC samples prepared at three different concentration levels (low, mid, and high) and analyzed along with calibration standards.

f. Precision:

The precision of a DBS LC‐MS/MS method was evaluated using QC samples prepared at three different concentration levels (low, medium, and high) and analyzed along with calibration standards. The intra‐ (within) day precision was determined by analyzing 15 replicates of three levels of QC samples. Inter‐ (between) day precision is determined by analyzing three levels of QC samples over five different days with calibration standards.

g. Carry‐over:

Carry‐over was evaluated by injecting two solvent blank samples sequentially immediately after the highest calibration standard sample injection.

h. Correlation:

The clinical correlation between testosterone concentrations measured in DBS and venous serum samples was evaluated using samples from 20 individuals. Venous blood was collected in BD Vacutainer SSTTM II Advance tubes and EDTA tubes, and the DBS samples were generated by spotting 20 µL of EDTA whole blood onto DBS cards. Hematocrit (HCT) values were measured for each blood sample using a Sysmex XN‐1000 automated hematology analyzer. Serum testosterone concentrations were analyzed using an immunologic method, and the DBS concentrations were corrected for HCT using the equation: Corrected concentration = (DBS concentration)/(1 ‐ HCT) [14].

#### 2.10.1 Validation Methods of Performance Related Specifically to DBS

- Effect of HCT:

QC samples were prepared at low and high testosterone concentrations using human blood with various HCT values (0.30, 0.40, and 0.80). The samples were analyzed in duplicate. The measured testosterone concentration results were compared with that obtained from the DBS samples with an HCT of 0.40 (similar to the target patient population of 0.40).

b. Influence of Blood Spot Volume:

Different blood volumes spotted on DBS card/paper may result in different measured analyte concentrations from a fixed punch size and HCT value. QC samples at two concentration levels (e.g., low and high) were spotted on the paper/card in six replicates with three increasing volumes (e.g., 10, 20, and 60 µL). After drying, a single punch (e.g., 3 mm) was taken from the center of each DBS sample and analyzed along with a set of calibration standards, for which the spotted blood volume could be the same as one of the above QCs, or at a different volume.

c. Matrix Effect And Recovery:

The matrix effect in MS/MS detection and assay recovery was evaluated by preparing three extracts or solutions:

- DBS QC (low 0.1 ng/uL and high 10.0 ng/uL) sample extracts in three replicates;
- blank DBS sample extracts (at least in three replicates) post‐fortified with both the analyte and the internal standard with the concentrations the same as in A; and
- neat solutions in at least three replicates with the concentrations of the analytes and the internal standard the same as in A and B.

All the above extracts or solutions were assayed using LC‐MS/MS with a set of calibration standards.

d. Stability of the DBS:

A DBS sample was collected from a volunteer, dried at room temperature, and ran on LC/MS/MS to establish baseline results. Two more samples were collected from the same subject. One was stored at room temperature and one was refrigerated. Both stored samples were analyzed a week and a month later and compared to the baseline result.

e. Collection Method Verification:

Ten DBS samples were collected from volunteers and analyzed with LC/MS/MS for baseline results. The same volunteers received a take‐home collection kit and mailed their DBS cards by mail to be analyzed and compared to the on‐site collected sample.

## 3 RESULTS

**FIGURE 2:**
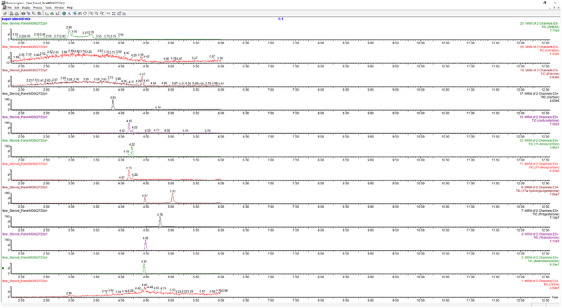
Chromatographic separation of testosterone and 11 steroid hormones assessed for potential coelution interference. Each chromatogram represents the retention time of one compound, showing no overlap between testosterone and other steroids.

**FIGURE 3:**
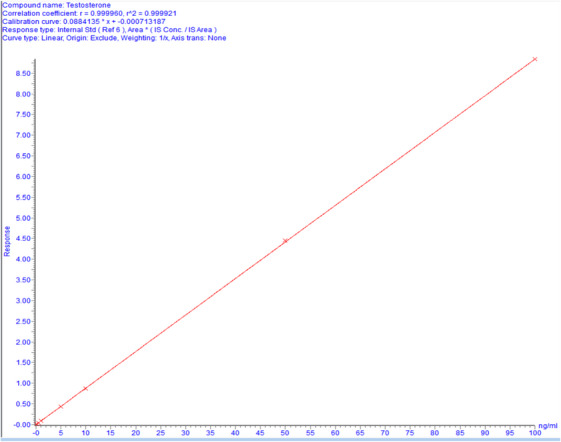
Calibration curve for testosterone assay showing linearity over a concentration range of 0.1–100 ng/mL. The correlation coefficient (r^2^) of 0.9992 indicates excellent linearity.

i. Interference:

None of the tested compounds showed coelution with testosterone due to a unique retention time and fragment mass combination (Figure 2).

j. Measuring Range:

The reportable range was determined to be 0.1–100 ng/mL (Figure 3).

k. Lower LOQ:

The LLOQ was determined to be 0.086 ng/mL, as calculated from the data in Table 1.

l. Limit of Detection:

**TABLE 1:** Data of dried blood spot (DBS) blank samples for the limit of detection (LOD) and lower limit of quantification (LLOQ) determination.

Sample name	Result (ng/mL)
DBS Blank	0.048
DBS Blank	0.044
DBS Blank	0.047
DBS Blank	0.042
DBS Blank	0.044
DBS Blank	0.043
DBS Blank	0.051
DBS Blank	0.052
DBS Blank	0.038
DBS Blank	0.049
Mean	0.046
SD	0.004
%CV	8.7%

The LOD was determined to be 0.058 ng/mL, as calculated from the data in Table 1.

m. Accuracy:

The accuracy, that is, the bias (%) from the nominal concentration values, was within ±20.

n. Precision:

The intra‐ and inter‐day precision, assessed by the standard deviation divided by the mean (CV%) from the replicate analysis, was <10%, as demonstrated in Tables 2 and 3.

**TABLE 2:** Inter‐day accuracy and precision data based on mean testosterone concentrations over 5 days.

	Inter‐day precision (using means)		
	Low (1 ng/mL)	Med (10 ng/mL)	High (50 ng/mL)
Day 1	1.08	10.44	46.29
Day 2	1.14	10.37	43.99
Day 3	1.27	10.01	40.65
Day 4	1.11	10.13	42.71
Day 5	1.16	10.78	43.69
Mean	1.15	10.35	43.46
SD	0.07	0.27	1.84
%CV	5.2%	2.6%	4.2%

**TABLE 3:** Intra‐day accuracy and precision data for testosterone assay at low, medium, and high concentration levels.

	Low			Med			High	
	Sample Conc	Result (ng/mL)		Sample Conc	Result (ng/mL)		Sample Conc	Result (ng/mL)
1	1 ng/mL	1.16	1	10 ng/mL	9.86	1	50 ng/mL	43.47
2	1 ng/mL	1.22	2	10 ng/mL	10.78	2	50 ng/mL	46.56
3	1 ng/mL	1.17	3	10 ng/mL	11.62	3	50 ng/mL	45.97
4	1 ng/mL	1.13	4	10 ng/mL	10.31	4	50 ng/mL	42.76
5	1 ng/mL	1.14	5	10 ng/mL	10.55	5	50 ng/mL	44.77
6	1 ng/mL	1.24	6	10 ng/mL	10.88	6	50 ng/mL	44.64
7	1 ng/mL	1.12	7	10 ng/mL	10.74	7	50 ng/mL	46.22
8	1 ng/mL	1.18	8	10 ng/mL	10.51	8	50 ng/mL	45.43
9	1 ng/mL	1.11	9	10 ng/mL	10.53	9	50 ng/mL	43.62
10	1 ng/mL	1.16	10	10 ng/mL	10.42	10	50 ng/mL	43.87
11	1 ng/mL	1.17	11	10 ng/mL	10.72	11	50 ng/mL	46.92
12	1 ng/mL	1.13	12	10 ng/mL	11.05	12	50 ng/mL	43.72
13	1 ng/mL	1.09	13	10 ng/mL	10.21	13	50 ng/mL	42.53
14	1 ng/mL	1.28	14	10 ng/mL	10.92	14	50 ng/mL	45.10
15	1 ng/mL	1.13	15	10 ng/mL	10.86	15	50 ng/mL	44.57
	Mean	1.16		Mean	10.66		Mean	44.68
	SD	0.05		SD	0.39		SD	1.31
	%CV	3.4%		%CV	3.7%		%CV	2.9%

o. Carry‐over:

As required, the response in the first blank matrix injection at the retention time of the analyte or internal standard was less than 20% of the mean response of an LLOQ sample for the analyte and less than 5% of the mean response for the internal standard.

p. Correlation:

A greater than 90% correlation between the serum and corrected DBS testosterone levels was observed with a Spearman's correlation coefficient (r^2^) of 0.988 (Figure 4).

**FIGURE 4:**
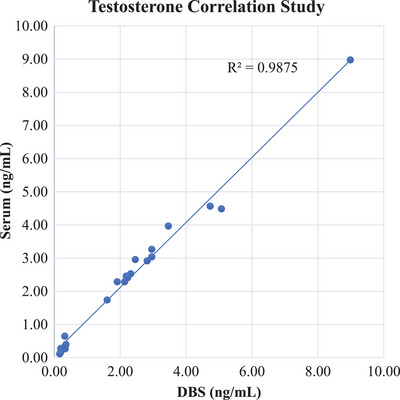
Correlation between testosterone concentrations measured in DBS and venous serum samples. The plot demonstrates a strong positive correlation between the two methods, with an R^2^ value of 0.9875, indicating high agreement between DBS and serum testosterone levels.

### 3.1 Validation of Specific Performance Data Related Specifically to DBS Yielded the Following Results

**FIGURE 5:**
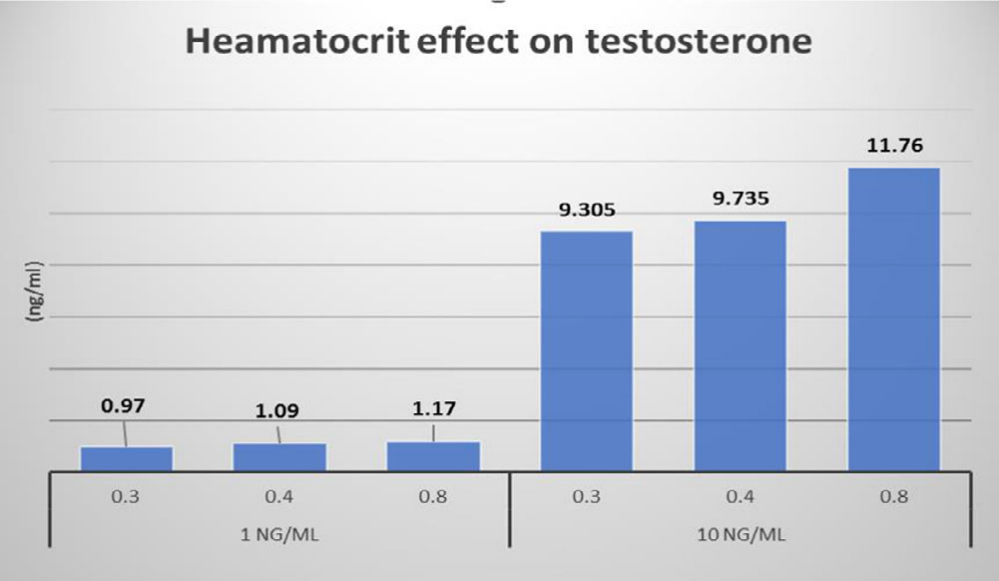
Effect of hematocrit on testosterone quantification in dried blood spot (DBS) samples at 1 and 10 ng/mL.

**FIGURE 6:**
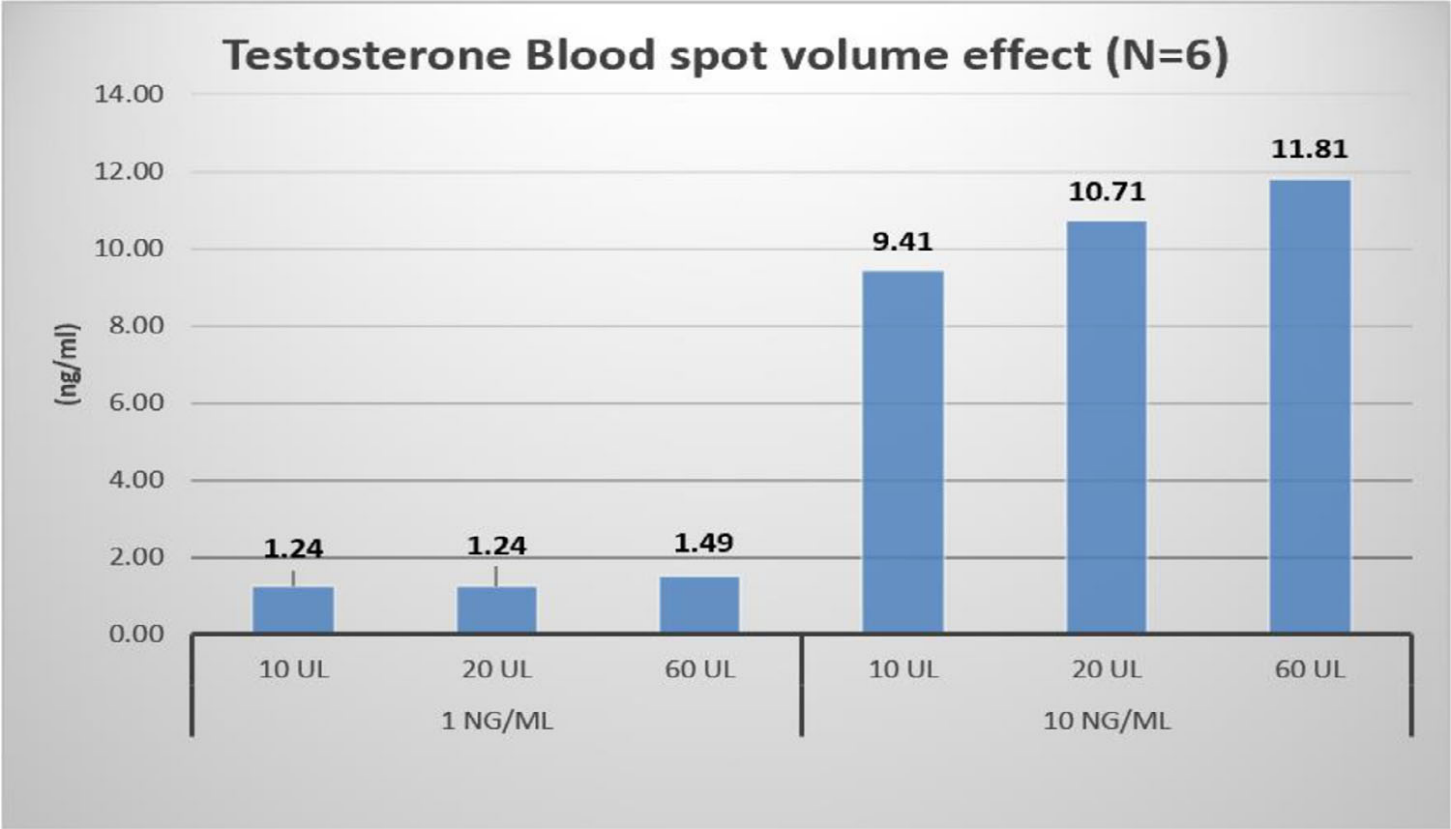
Blood spot volume effect on testosterone quantification in dried blood spot (DBS) samples at 1 and 10 ng/mL.

f. Effect of HCT (HT):

An apparent (±20%) impact of HT was observed on the quantification of testosterone (Figure 5).

g. Influence of Blood Spot Volume:

An apparent (±20%) volume effect was observed (Figure 6).

h. Matrix Effect and Recovery:

Matrix effect at 1 ng/mL = 3.65%, Recovery at 1 ng/mL = 75%

Matrix effect at 10 ng/mL = 0.77%, Recovery at 10 ng/mL = 81%

i. Stability of the DBS:

The sample was stable for up to a month both at room temperature and 2–8°C, as shown in Table 4.

**TABLE 4:** Stability of testosterone in dried blood spot (DBS) samples over time.

	Baseline (ng/mL)	Day 8 (ng/mL)	Day 30 (ng/mL)
Room temperature	1.732	1.793	1.777
Refrigerated	1.732	1.787	1.79

j. Collection method verification:

The results correlated well with a correlation factor of .8 using the correl formula.

## 4 DISCUSSION

The findings from the validation of the testosterone assay demonstrate that the method is robust, accurate, and precise across a wide range of conditions. The assay showed excellent linearity with a correlation coefficient (r^2^) of 0.999 across testosterone concentrations ranging from 0.1 to 100 ng/mL. The evaluation of inter‐ and intra‐day precision further supports the robustness of the assay. Inter‐day precision, assessed over 5 days at low, medium, and high testosterone concentrations, demonstrated %CV values below 10% for all concentrations. Similarly, the intra‐day precision evaluation showed %CV values below 5%, further indicating the reliability of the method in providing consistent results within a single day.

The LLOQ and LOD were calculated at 0.086 and 0.058 ng/mL, respectively, confirming the assay's sensitivity in detecting low testosterone concentrations. These results are aligned with established validation guidelines, ensuring the method's suitability for clinical and research applications where accurate testosterone quantification at low concentrations is required. Matrix effect and recovery assessments confirmed that the method is resistant to matrix effects, with acceptable recoveries of 75% at low concentrations and 81% at high concentrations.

Clinical correlation analysis revealed a strong agreement between testosterone concentrations measured in DBS and venous serum samples, with a correlation coefficient (r^2^) of 0.988. This indicates that DBS can serve as a reliable surrogate for serum testosterone measurements, providing a less invasive and more convenient method for clinical testosterone monitoring​. The stability of testosterone in DBS was confirmed under various storage conditions, with samples remaining stable for up to 30 days under both room temperature and refrigerated conditions. This stability study is consistent with findings from other studies on steroid hormones, such as those reported by Grecsó et al. [7], which demonstrated similar stability patterns for other steroid hormones in DBS. Additionally, the interference study demonstrated that none of the 11 tested steroid hormones coeluted with testosterone. Each hormone exhibited distinct retention times and unique fragment masses, ensuring the specificity of the testosterone assay in the presence of these structurally similar compounds. The validation results are summarized in Table 5.

**TABLE 5:** Summary of testosterone assay validation results.

Study	Result
Interference	Negative
Measuring range	0.1–100 ng/mL
LLOQ	0.086 ng/mL
LOD	0.058 ng/mL
Accuracy	±20%
Precision	<10%
Carry‐over	Negative
Correlation	>90%
Effect of hematocrit	(+/−20%)
*Matrix Effect*:	
Matrix effect at 1 ng/mL	3.65%
Matrix effect at 10 ng/mL	0.77%
*Recovery*:	
Recovery at 1 ng/mL	75%
Recovery at 10 ng/mL	81%
Stability of the DBS	Month at 2–25°C
Collection method verification	correlation coefficient = 0.8

Overall, the testosterone assay validated here exhibits high accuracy, precision, and stability, with minimal matrix effects or interference. It is suitable for use in clinical and research applications where reliable routine testing of testosterone quantification is essential.

## Conflicts of Interest

The author declares no conflicts of interest.

## Supporting information



Supporting Information

## Data Availability

The data that support the findings of this study are available from the corresponding author upon reasonable request.
