# Supplementary material for: Validation of a Dried Blood Spot Assay for Testosterone Measurement Using Liquid Chromatography‐Tandem Mass Spectrometry
Source: Anal Sci Adv. 2024 Nov 2;5(11-12):e202400035. doi: 10.1002/ansa.202400035 (PMC11627179; doi:10.1002/ansa.202400035)
Supplement: Supplementary file 1 — Supporting Information [file ANSA-5-e202400035-s001.docx]

**Supplementary file for materials and methods:**

**Key instruments:**

Table S1: Details on the LC-MS/MS system

|  | Make | Model | S/N |
| --- | --- | --- | --- |
| Waters Xevo TQ-XS MS Detector | Waters Corp. | Xevo TQ-XS | WBA1351 |
| Column Manager | Waters Corp. |  | F21CMP250G |
| UPLC Column | Waters Corp. | Acquity UPLC HSS T3 1.8 um | 2.4832E+12 |
| Binary Solvent Manager (BSM) | Waters Corp. |  | G21BUR180M |
| Sample Manager | Waters Corp. |  | E21USM147M |
| Sample Organizer | Waters Corp. |  | C21CPO186H |

Table S2: Details on the auxiliary instruments

|  | Make | Model | S/N |
| --- | --- | --- | --- |
| DBS Puncher | Perkin Elmer |  |  |
| Nitrogen Generator | Peak Scientific | Genius XE35-120V | 721020025 |
| Heated microplate vortexer | Benchmark scientific In | H6002 | 020-16031-21030006 |
| Electronic Scale | Hanchen | BSM 120.4 | N9210611-058 |
| Centrifuge | Benchmark scientific In | Swing 60 WCC | Sprint 6 - 00057 |
| Sonicator | Co-Z | 60A | ROHS2111 |
| Heating Plate with 96 gas needles | BT Lab Systems | BT1604 | BL-E21-1033 |
| Nitrogen Gas Tank |  |  |  |
| Argon Gas Tank |  |  |  |

Table S3: List of consumables

|  | Vendor | Part Number |
| --- | --- | --- |
| 226 Spot Saver RUO Card | PerkinElmer | GR2261005 |
| 2-mL deep 96-well plate | Thermo Fisher | 95040450 |
| 1-mL deep 96-well plate | Nest | 502162 |
| Adhesive Seals |  |  |
| 6-mL transfer pipettes | Grainger | 21F235 |
| 1250-uL pipette tips | Integra | 4445 |
| 125 uL pipette tips | Integra | 4425 |
| 20 uL pipette tips | Integra | 6455 |
| 2-mL Eppendorf tubes | Sigma Aldrich | Z628034-500EA |
| 15 mL conical tubes | Thermo Scientific | 12-565-269 |

Table S4: Details on the reagents & chemicals

|  | Type | Chemical Formula | Part # | Storage |
| --- | --- | --- | --- | --- |
| Testosterone  1.0 mg/mL | Standard | C_19_H_28_O_2_ | Cerilliant: T-037 | -20 C^0^ |
| Testosterone-2,3,4-13C3  100 ug/mL | Internal Standard | ^13^C_3_C_16_H_28_O_2_ | Cerilliant: T-070 | -20 C^0^ |
| Mass Spec Gold Human Serum, Ultra-Low Hormones & Steroids |  | ___ | Golden West Diagnostics: MSG3000 | -20 C0 |
| Ammonium Fluoride | Mobile phase | NH4F | Sigma Aldrich: 338869 | RT |
| LC/MS grade Methanol | Solvent/Mobile phase | MeOH | Thermo Fisher: A456-4 | RT |

**Sample preparation (A-E):**

1. Testosterone standard solution
2. Testosterone internal standard solution
3. Steroid-free whole blood
4. Testosterone standard DBS
5. Mobile phases and maintenance solutions
6. **Preparation of testosterone standard solution**

The testosterone standard solution was prepared and used to spike steroid-free blood to create the calibration curve and QC material. Once prepared, the testosterone standard solution was stored at -20°C. The following procedure was used to make intermediate (1000X) and intermediate working (10X) dilutions of the testosterone standard:

1. An intermediate dilution of 10 µg/mL (1000X) was prepared by mixing 990 µL of LC-MS grade methanol with 10 µL of testosterone standard (1 mg/mL, Cerilliant, USA) in a 2 mL tube.
2. In a separate 2 mL tube, 990 µL of LC-MS grade methanol was mixed with 10 µL of the 1000X dilution to make a 100 ng/mL (10X) dilution, which was then used to spike steroid-free blood.

|  | Concentration  (ng/ml) | Mix |
| --- | --- | --- |
| Intermediate (1000X) | 10000 | 990 ul of LC-MS grade methanol + 10 ul of testosterone standard (1 mg/ml) |
| Intermediate working  (10X) | 100 | 990 ul of steroid free blood + 10 ul of intermediate (1000X) testosterone standard dilution |

1. **Preparation of testosterone internal standard (IS) solution**

The testosterone internal standard contains a Carbon-13 labeled testosterone molecule with a similar retention time on LC but differing masses on MS, allowing it to evaluate the extraction recovery of each sample individually. The internal standard solution was prepared on a weekly basis and stored at -20°C. The following procedure was used to make intermediate (100X), intermediate working (10X), and working (1X) testosterone IS dilutions of the testosterone internal standard:

1. An intermediate dilution of 5 µg/mL (100X) was prepared by mixing 950 µL of LC-MS grade methanol with 50 µL of Testosterone-2,3,4-13C3 100 µg/mL (Cerilliant, USA) in a labeled 2 mL tube.
2. In a separate 2 mL tube, 900 µL of LC-MS grade methanol was mixed with 100 µL of the intermediate (100X) testosterone IS dilution to make a 500 ng/mL intermediate working (10X) dilution.
3. Finally, ten 2 mL tubes labeled "IS" were prepared, and the final working dilution of 50 ng/mL (1X) was made by mixing 900 µL of LC-MS grade methanol with 100 µL of the intermediate working (10X) testosterone IS dilution in each tube.

|  | Concentration  (ng/mL) | Mix |
| --- | --- | --- |
| Intermediate (100X) | 5000 ng/mL | 950 uL of LC-MS methanol + 50 uL of Testosterone-2,3,4-13C3 (100 ug/mL) |
| Intermediate working (10X) | 500 ng/mL | 900 uL of LC-MS methanol + 100 uL of intermediate (100x) testosterone IS dilution |
| Working Dilution (1x) | 50 ng/mL | 900 uL LC-MS methanol + 100 uL of intermediate working (10x) testosterone IS dilution |

*Note: ensure to vortex and spin in between each dilution

**C. Preparation of steroid-free whole blood (Blank Standard)**

Calibration standards and QC samples were prepared using commercially available stripped serum and washed red blood cells (RBCs) from healthy volunteers, following a modified method reported by Higashi et al. (Higashi et al., 2008). The following procedure was used to wash the blood and prepare steroid-free blood with a 40% hematocrit ratio:

1. Whole blood was collected from volunteers via venipuncture in lavender top tubes and delivered to lab personnel.
2. The lavender tubes containing whole blood were centrifuged at 1500 × g for 15 minutes. The plasma layer (top) was aspirated and discarded using a 6 mL transfer pipette.
3. An equal amount of the remaining RBCs in each tube was transferred into phosphate buffer saline (PBS) using another transfer pipette and inverted until the blood was homogenized.
4. The lavender tubes (now containing PBS) were centrifuged again at 1500 × g for another 15 minutes, and the top PBS layer was discarded.
5. Steps 3 and 4 were repeated for a total of three PBS washes.
6. Finally, after discarding the PBS from the third wash, the remaining blood cells were transferred to a new 15 mL conical tube and mixed with steroid-free serum (Golden West, USA):
   - 600 µL of serum was added for every 400 µL of blood cells.

**D. Preparation of testosterone standard DBS (calibration standards and QC)**

Calibration standards and quality control (QC) samples were prepared by spiking steroid-free whole blood with testosterone standard solution at known concentrations. Eight calibration standards (Blank, zero, and six calibrators) were prepared in blood, then spotted onto DBS cards and dried. DBS calibrators were prepared using the same batch of blood and serially diluted. Calibrator 8 was prepared first, and it was then serially diluted to create the lower calibrators in separate tubes. The following chart was used to prepare testosterone standard DBS (calibrators 3–8):

|  | Concentration  (ng/mL) | Mix |
| --- | --- | --- |
| Cal 8 | 10 | 900 uL of steroid free blood + 100 of intermediate working (10x) testosterone standard dilution |
| Cal 7 | 5 | 500 uL of steroid free blood + 500 of (Cal 8) |
| Cal 6 | 2 | 600 uL of steroid free blood + 400 of (Cal 7) |
| Cal 5 | 0.4 | 800 uL of steroid free blood + 200 of (Cal 6) |
| Cal 4 | 0.16 | 600 uL of steroid free blood + 400 of (Cal 5) |
| Cal 3 | 0.08 | 500 uL of steroid free blood + 500 of (Cal 4) |
| Blank | 0 | 1000 uL of steroid free blood |

*Note: ensure to vortex and spin in between each dilution

For QC, a separate batch of steroid-free blood and testosterone standard solution was used. The testosterone standard solution was prepared at similar concentrations to those used for the calibration curve. The following chart was used to prepare testosterone QC DBS (LLOQ, Low, Medium, and High):

|  | Concentration  (ng/mL) | Mix |
| --- | --- | --- |
| High | 5 | 950 uL of steroid free blood + 50 of intermediate working (10x) testosterone standard dilution |
| Med | 1 | 800 uL of steroid free blood + 200 of (High) |
| Low | 0.2 | 800 uL of steroid free blood + 200 of (Med) |
| LLOQ | 0.08 | 600 uL of steroid free blood + 400 of (Low) |

*Note: ensure to vortex and spin in between each dilution

After making the dilutions for both QC and Calibration curve, use a multichannel pipette to transfer testosterone standard solution to labeled DBS cards and allow to dry for > 3 hours. Each batch of QC and calibration curve must be tested separately before using with patient samples.

**E. Preparation of mobile phases and maintenance solutions**

1. 5M Ammonium Fluoride Solution: Weighed 185 mg of ammonium fluoride (99.9+%) was added to a 2 mL HPLC vial. Then, 1 mL of deionized water was added, and the solution was shaken well.
2. Mobile Phase A: 20 µL of the 5M ammonium fluoride solution was pipetted into one liter of deionized water and mixed well.
3. Mobile Phase B and Wash Solvent: Pure LC-MS grade Methanol
4. Purge Solvent and Seal Wash: 100 mL of LC-MS grade methanol was added to 900 mL of deionized water and mixed well.

**Operational procedures:**

LC-MS/MS workflow can be broken down into the following steps:

1. Extraction
2. Instrument start up
3. Injection
4. Result analysis
5. Result Reporting
   - 1. **DBS extraction protocol**
6. All samples were checked to ensure they were acceptable and properly labeled.
7. Using the PerkinElmer DBS puncher, three 3 mm punches were placed in each specimen, preferably from a single circle.
8. 10 µL of steroids internal standard solution (10 ng/mL, 1X) was added to each sample well (including the calibration curve and QC).
9. 500 µL of LC-MS grade methanol was added to each sample well, and the plate was sealed using adhesive film.
10. The plate was shaken for 5 minutes at a speed of 1000 RPM.
11. The plate was sonicated for 20 minutes at 30°C, ensuring that half the plate was submerged in water.
12. The 96-well, 1 mL plate was centrifuged at 2000 × g for 2 minutes.
13. The supernatants were transferred to a separate 96-well, 1 mL plate.
14. The 1 mL plate was placed on a heated plate and evaporated to dryness under a gentle stream of nitrogen at 60°C.
15. Once completely dry, each sample well was reconstituted with 100 µL of a methanol/water (50:50, v/v) solution and shaken for 10 minutes at a speed of 1000 RPM.
16. The 96-well, 1 mL plate was centrifuged at 2000 × g for 2 minutes.
17. The plate was sealed using adhesive film, and the injection procedure was initiated.
    - 1. **Instrument start up**
18. The API gas was turned on, and the mass spectrometer was put into ‘operate’ mode.
19. The flow was primed and started: i. The "MassLynx" software was opened. ii. The "Status" tab was clicked. iii. The "Inlet Method" tab from the top left corner was selected. iv. The “Binary Solvent” box was right-clicked, and "Start-up system" was selected to prime the mobile phase tubes and initiate the flow at 0.1 mL/min.
20. The flow rate was gradually increased to 0.8 mL/min: i. From the "Inlet Method" screen, the flow rate was increased first to 0.5 mL/min and then to 0.8 mL/min while monitoring the pressure. ii. It was ensured that the systemic pressure remained below 13,000 PSI at 0.8 mL/min. iii. The instrument was allowed sufficient time to equilibrate, ensuring the systemic pressure was steady for at least two minutes before proceeding with injection.
21. From the “Status” tab, the “MS Console” tab was opened, and the gas flow and operator mode were turned on: i. The system status displayed a green ready icon afterward.


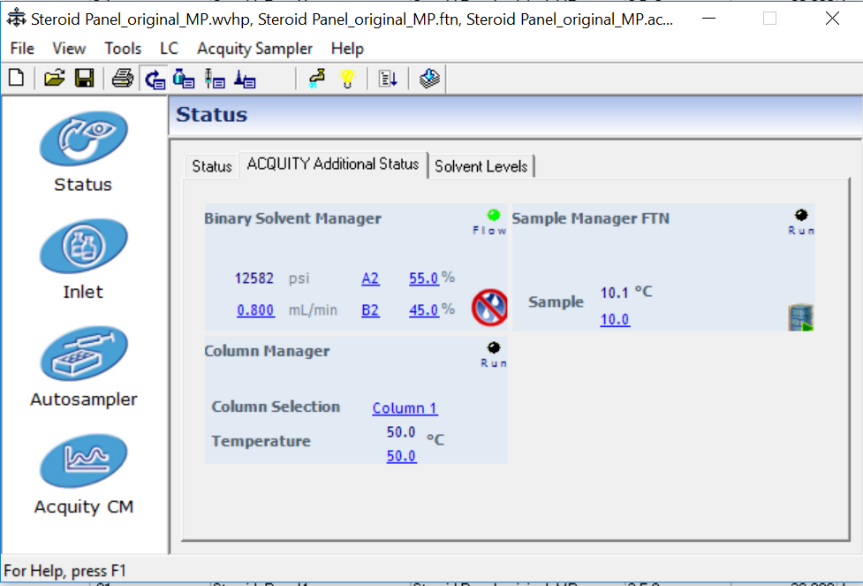


**Supplementary figure 1.** LC-MS/MS System Status During Steroid Panel Analysis. The system shows a flow rate of 0.800 mL/min with 55% solvent A2 and 45% solvent B2 at 12,582 psi. The column temperature is set to 50.0°C, and the sample is held at 10.1°C. The system is in "Run" mode with sample well "10.0. "

**HPLC conditions:**

| Instrument: | Waters Xevo I-Class TQ XS UPLC/MS/MS system |
| --- | --- |
| Ionization mode: | Electrospray positive ion; MRM mode |
| Column: | Acquity HSS T3 3.0x50 mm 1.8µm |
| Mobile Phase A: | H2O+10 µM ammonium fluoride |
| Mobile Phase B: | LC-MS grade methanol |
| Flow Rate: | 0.8 mL/min |
| Injection volume | 30 µl |
| Sample Compartment Temp: | 15°C |
| Column Temperature: | 50°C |
| Wash Solvent: | LC-MS grade methanol |
| Purge solvent/Seal Wash: | 90:10 water/methanol |
| MS Detection: | MRM (see table 1) |
| Run Time: | 9 minutes |

**Gradient:**

| Time, min | % A | % B | Curve |
| --- | --- | --- | --- |
| 1.0 | 55.0 | 45.0 | 6 |
| 2.5 | 47.5 | 52.5 | 6 |
| 3.5 | 47.5 | 52.8 | 6 |
| 5.0 | 35.0 | 65.0 | 6 |
| 6.25 | 10.0 | 90.0 | 6 |
| 7.0 | 55.0 | 45.0 | 11 |
| 9.0 | 55.0 | 45.0 | 11 |

**MS conditions:**

| Capillary Voltage (KV): | 1.0 |
| --- | --- |
| Cone Voltage: | refer to MRM Transition table |
| Source Temperature: | 150 °C |
| Desolvation Temperature: | 500 °C |
| Desolvation Gas (L/hr): | 1000 |
| Cone Gas (l/hr): | 150 |
| Nebulizer Pressure (Bar): | 7 |

**MRM transitions:**

| Compound | Precursor | Production | Dwell Time, sec | Cone Voltage | CE |
| --- | --- | --- | --- | --- | --- |
| Testosterone 289.2 | 289.2 | 97.0 | 0.025 | 40 | 22 |
| Testosterone 289.2 | 289.2 | 109.0 | 0.025 | 40 | 26 |
| Testosterone-IS 292.3 | 292.3 | 100.4 | 0.025 | 42 | 20 |

**Mass spectrometric conditions:**

Acquisition mode: MRM

Ionization mode: ESI in the Positive Ion Mode

ESI Voltage: 5500V

Turbo Gas Temperature: 700 0C

Curtain Gas: Nitrogen 22 psi

Collision Gas: Nitrogen 5 psi

Ion Source Gas 1: Air 50 psi

Ion Source Gas 2: Air 70 psi

Declustering Potential: 96 V

Entrance Potential: 10 V

Collision Energy: 29 V

Collision Exit Potential: 12 V

SRM masses (m/z):

Analyte SRM

(m/z)

Transition

used for:

Expected Retention time (min) Testosterone: 289.4>97 Quantitation 4.6 min

289.4>109 Confirmation 4.6 min

13C3-Testosterone: 292.2>100 Quantitation 4.6 min

292.2>112 Confirmation 4.6 min

- - 1. **Injection procedure**

1. The plate was placed inside the sample organizer with “A1” facing inwards.
2. A new worksheet was created on MassLynx for each day: a. The “File” tab was clicked, then “New” was selected to create a new worksheet.
3. A right-click on any field allowed for the addition of more rows by selecting “Add.”
4. The appropriate method was selected: a. MS Method: Steroid_Testosterone_289.4 b. UPLC Method: Steroid Panel original MP c. Injection Volume: 30 µL
5. Sample names and well locations were assigned: a. The “Vial/Well” field was double-clicked to assign the well location. b. The “Sample Name” field was used to assign a sample name.
6. The sample type was assigned: a. Blank for Calibrator 1. b. Standard for Calibrators 2–8. c. QC for quality control samples. d. Analyte for patient samples.
7. Once all changes were saved, the “Play” icon was clicked to queue the samples for injection.


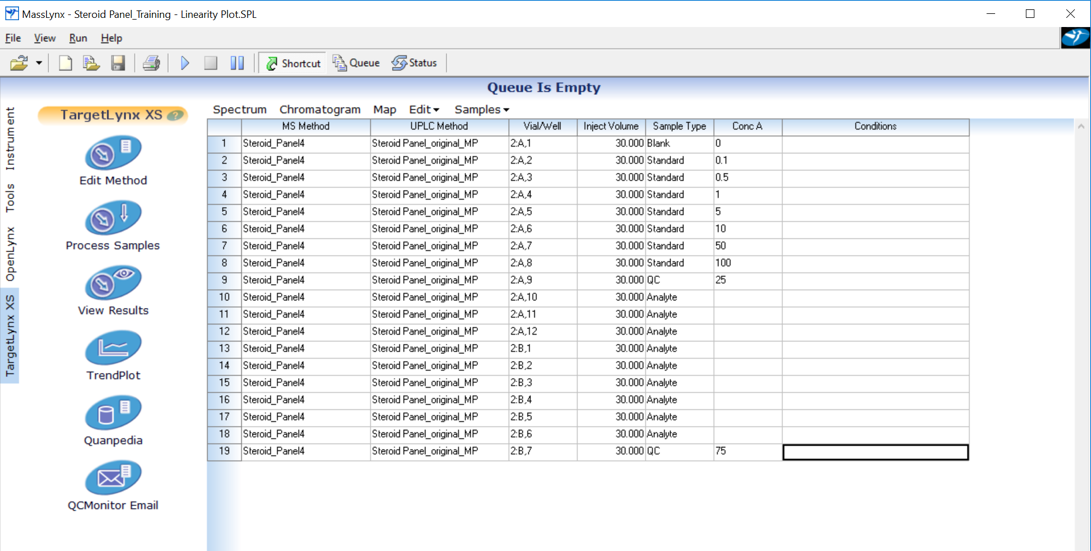


**Supplementary figure 2.** Queue Setup for Steroid Panel Analysis in MassLynx. MassLynx interface showing the queued steroid panel samples with vial/well numbers, methods, injection volumes (30 µL), sample types, and concentrations.

- - 1. **Results processing using TargetLynx:**

1. Once all samples had been injected, all rows of the worksheet were highlighted, and the results were processed via TargetLynx: a. The “Shortcut” button at the top of the page was clicked. b. “TargetLynx” was selected from the left menu. c. “Process Samples” was clicked.
2. It was ensured that the calibration curve passed, behaving in a linear fashion with r² > 0.99 (see the figure below): a. In the case of an outlier point, the sample row was double-clicked from the summary bar, and proper peak integration for both testosterone and testosterone IS was manually inspected. b. To manually adjust the integration, the correct peak was right-clicked, “Add Peak” was selected, and the changes were saved. c. To remove a point from the calibration curve, the sample row was right-clicked from the summary bar, and “Exclude” was selected.
3. Proper integration of each patient sample was manually inspected and adjusted if necessary.
4. After fully analyzing the run, “File” was clicked, followed by “Save As,” to save the worksheet.
5. Finally, “File” was clicked again, followed by “Export,” and “Current Summary” was selected to export the results into an Excel file for the LIS pick-up location.


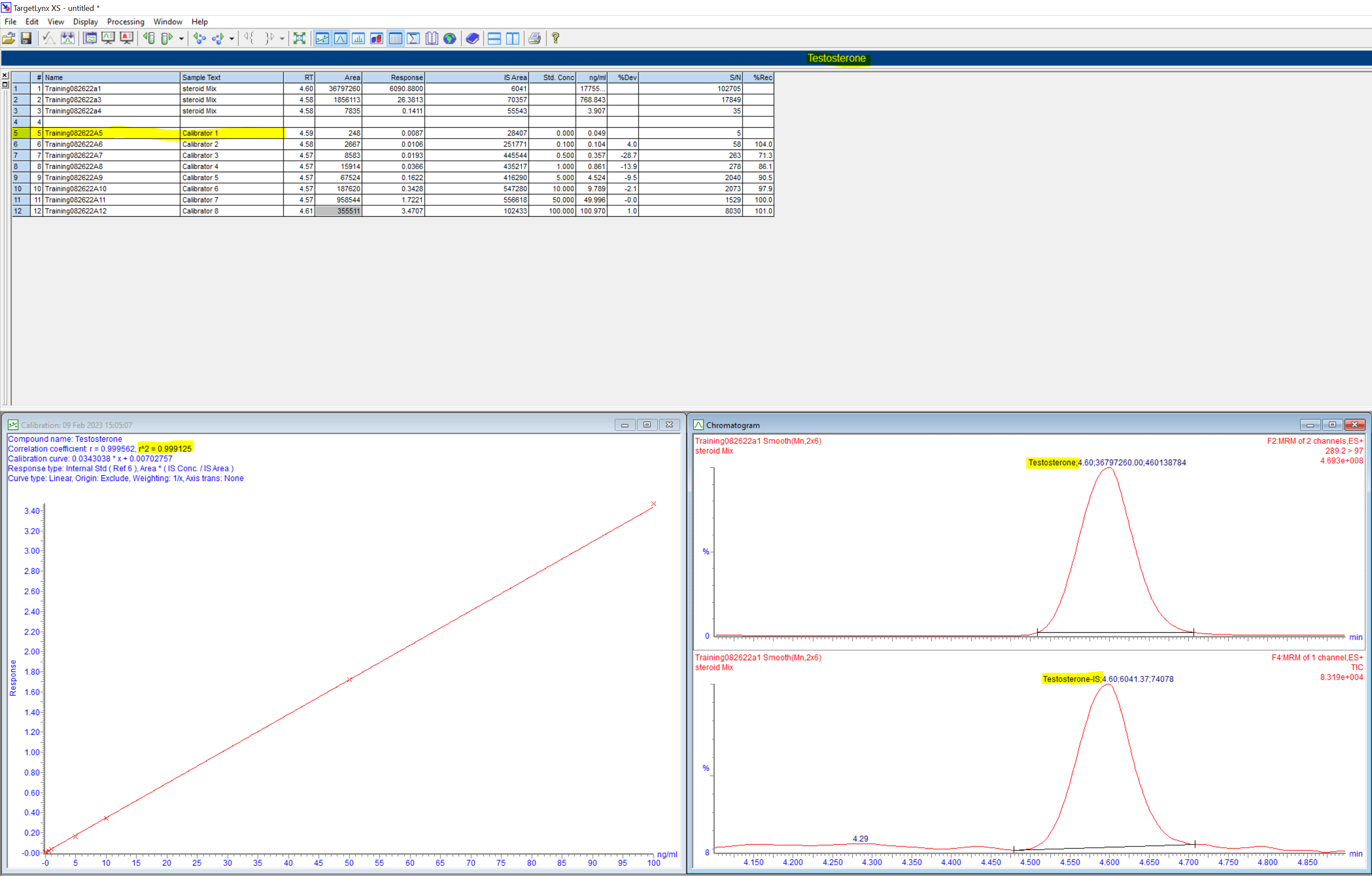


**Supplementary figure 3.** Calibration Curve and Testosterone Chromatogram. Calibration curve with linear regression (r² > 0.99) and chromatograms showing testosterone peaks at 4.40 minutes.

**Results interpretation:**

**Calibration and quality control:**

1. Each run included an eight-point calibration curve (blank, zero, plus six calibrators) ranging from 0 to 10 ng/mL, along with three QC points (low, medium, and high) and an additional QC point followed by a blank after every 10 samples.
2. Calibration curves were generated using the area ratios from the calibrators and their assigned values through ordinary linear regression. A coefficient value (r²) of >0.98 was required to pass the run. Sample batches with invalid calibration curves were not processed further.
3. All calibration standards were required to be within ±20% of their assigned nominal values.
4. For quality control, two out of three QC points needed to be between 80-120% of their assigned nominal values.
5. Area ratios were calculated from the quantitation ion and the confirmation ion (the "Confirmation Ion Ratio"). Only analytes that showed a Confirmation Ion Ratio within ±15% of the target value were considered for further processing.

**Valid Result:** A patient sample was considered valid if it met all the following criteria:

1. Had a signal within the predicted retention time of 4.6 min (±0.25 min).
2. The internal standard signal was comparable to the calibration curve (<15% deviance).
3. The result fell within the reportable range of 0.08 to 10 ng/mL.

**Inconclusive Result:** A patient sample was considered invalid if it met any of the following criteria:

1. Lacked a signal within the predicted retention time of 4.6 min (±0.25 min).
2. The internal standard signal was not comparable to the calibration curve (>15% deviance).
3. The result was below the LLOQ or outside the reportable range of 0.08 to 10 ng/mL.
